# A Phylogenetic Analysis of 34 Chloroplast Genomes Elucidates the Relationships between Wild and Domestic Species within the Genus *Citrus*

**DOI:** 10.1093/molbev/msv082

**Published:** 2015-04-14

**Authors:** Jose Carbonell-Caballero, Roberto Alonso, Victoria Ibañez, Javier Terol, Manuel Talon, Joaquin Dopazo

**Affiliations:** ^1^Computational Genomics Department, Centro de Investigación Príncipe Felipe (CIPF), Valencia, Spain; ^2^Centro de Genómica, Instituto Valenciano de Investigaciones Agrarias, Moncada, Valencia, Spain; ^3^Functional Genomics Node, Spanish National Institute of Bioinformatics at CIPF, Valencia, Spain

**Keywords:** chloroplast genome, citrus, phylogeny, heteroplasmy, selection

## Abstract

*Citrus* genus includes some of the most important cultivated fruit trees worldwide. Despite being extensively studied because of its commercial relevance, the origin of cultivated citrus species and the history of its domestication still remain an open question. Here, we present a phylogenetic analysis of the chloroplast genomes of 34 citrus genotypes which constitutes the most comprehensive and detailed study to date on the evolution and variability of the genus *Citrus*. A statistical model was used to estimate divergence times between the major citrus groups. Additionally, a complete map of the variability across the genome of different citrus species was produced, including single nucleotide variants, heteroplasmic positions, indels (insertions and deletions), and large structural variants. The distribution of all these variants provided further independent support to the phylogeny obtained. An unexpected finding was the high level of heteroplasmy found in several of the analyzed genomes. The use of the complete chloroplast DNA not only paves the way for a better understanding of the phylogenetic relationships within the *Citrus* genus but also provides original insights into other elusive evolutionary processes, such as chloroplast inheritance, heteroplasmy, and gene selection.

## Introduction

The genus *Citrus* comprises some of the most important cultivated fruit trees worldwide. In spite of the economical transcendence of mandarins, oranges, lemons, limes, grapefruits, and other popular citrus, the domestication process and the taxonomy of these species are still poorly understood.

The genus *Citrus* is included in the subfamily *Aurantioideae*, belonging to the family *Rutaceae*. This Family, widely distributed in the tropics, is composed of about 160 genera and 1,800 species characterized by peculiar foliar oil glands. Previous reports on the relationships within *Rutaceae* subfamily *Aurantioideae* estimated that *Citrus* diverged about 7 My (Pfeil and Crisp 2008). Phylogenetic studies (Scora 1975; Barrett and Rhodes 1976) agree that most cultivated citrus are derived through hybridization from three true citrus species: *Citrus reticulata* (mandarins), *C. maxima* (pummelos), and *C. medica* (citrons). Further studies supported this hypothesis and some of them have also added *C. micrantha* as a fourth foundational species (Nicolosi et al. 2000; Barkley et al. 2006; Garcia-Lor et al. 2012, 2013; Ollitrault et al. 2012). However, aside from this general agreement, there are still many discrepancies regarding the phylogenetic relations within the genus *Citrus* (Scora 1975; Pfeil and Crisp 2008; Bayer et al. 2009; Penjor et al. 2013).

The recent availability of nuclear (Xu et al. 2013; Wu et al. 2014) and chloroplast (Bausher et al. 2006) reference genomes of *Citrus* species enables more detailed studies of the origin, domestication, and phylogenetic relationships within this group. In particular, whole chloroplast genome analysis is known to provide high resolution plant phylogenies (Parks et al. 2009). Chloroplast genomes of plants are known to be highly conserved in both gene order and gene content (Raubeson and Jansen 2005). They exhibit a substitution rate much lower than nuclear DNA, which is even significantly reduced in the inverted repeat regions (Wolfe et al. 1987). The chloroplast genome sequence of *C. sinensis* is a nonrecombining circular unit of 160,129 bp length containing 133 genes (including 89 protein-coding, 4 rRNAs, and 30 distinct tRNAs) and a small number of large duplications, which is intervened by inverted repeat regions (Bausher et al. 2006).

In addition to their suitability for phylogenetic studies, complete chloroplast sequences can also provide insights into other elusive evolutionary processes, such as chloroplast inheritance, heteroplasmic phenomena, or gene selection. Heteroplasmy, defined as the presence of a mixture of organelle genomes (mitochondria or chloroplasts) within a cell or individual, is generally attributed to mutations or biparental inheritance and is visualized as a mechanism by which the organism can adapt rapidly. Although many angiosperms show maternal chloroplast inheritance, no less than one-third of them display biparental inheritance to some degree (Mogensen 1996). Heteroplasmy is expected to produce competition among the different cytoplasmic genomes, whereas the genes in the chloroplast can be subject to considerable selection both within and among individuals. Selective pressures along the evolution of the genus constitute another interesting aspect that can be studied in chloroplast genomes. Recent reports document significant biases of nonsynonymous over synonymous substitutions (positive selection) in several chloroplast genes. Thus, it has been demonstrated positive selection in *Flaveria* on both subunits of the chloroplast *rbcL* and nuclear *rbcS* genes encoding the large and small subunits of Rubisco (Kapralov et al. 2011). Moreover, high substitution evolutionary rates have also been reported for the chloroplast *clpP1* exon in three distantly related taxa (Erixon and Oxelman 2008).

Although previous studies on *Citrus* phylogenetic relationships were based on specific DNA markers or on partial cytoplasmic DNA sequences (Scora 1975; Barrett and Rhodes 1976; Pfeil and Crisp 2008; Bayer et al. 2009; Penjor et al. 2013), the analysis of the complete chloroplast genome clearly renders phylogenetic relationships with stronger support. In this work, 34 citrus chloroplast genomes representative of the genus *Citrus* have been sequenced and their sequences compared in order to construct the maternal phylogeny of most important citrus species. The reconstruction of the phylogeny enabled the study of positive selection signatures as well as the distribution of other mutational events, such as deletions, insertions, and larger structural variations (SVs) along the evolutionary history of the genus *Citrus*. Additionally, this comparative analysis also allowed new insights into divergence times between citrus species and revealed the existence of an unexpected level of heteroplasmy in citrus chloroplasts.

## Results

### Sequencing and Mapping

**Fig. 1.**
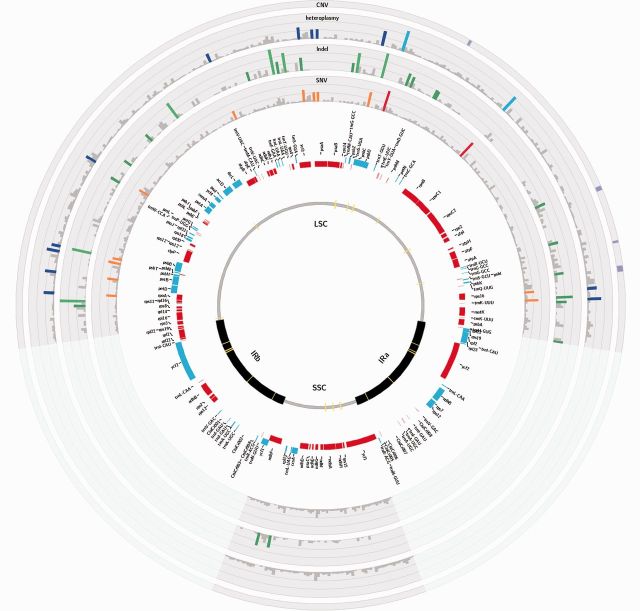
Variability of the genus *Citrus* represented over the Circular map of the *Citrus sinensis* chloroplast genome. The two inverted repeat regions (IRa and IRb) separate the large (LSC) and small (SSC) single copy regions, respectively. The yellow lines in the innermost circle correspond to regions with homology to nuclear sequences. Genes represented by blue rectangles are on positive strand, whereas genes represented by red rectangles are on negative strand. Density of SNVs and indels is represented by colored bars. Higher density of SNVs and indels is represented by darker bars. Heteroplasmy and large CNV are also depicted along the genome.

The chloroplast sequences of 34 citrus genotypes were obtained as described in Materials and Methods (sequence data were submitted to the ENA database with identifier ERP005411). The raw sequences were mapped onto the *C. sinensis* (Bausher et al. 2006) chloroplast genome as described in Materials and Methods. A total of 21 regions homologous to the nuclear DNA (accounting for 2% of the chloroplast genome size), along with the inverted repeat regions were removed from the mapping process (see fig. 1). Quality controls were acceptable for all the species analyzed. Coverage was very high (∼2,000×) and the resulting fraction of the analyzed genome with coverage over 15× was almost 100% (see table 1).

**Table 1.** Identity of the Chloroplast Genome of Several citrus and Citrus-Related Species Inferred from the Chloroplast DNA Sequence.

Species	Cultivar	Chloroplast Genome	SNVs	Indels	Mapped Reads	Mean Coverage	Genome Fraction > 15×
*Citrus sinensis*	Sweet orange	PU	0^a^	0^b^	4,794,814	2,097	100.0
*Citrus aurantium*	Sour orange	PU	16	04	20,959,168	9,078	100.0
*Citrus limon*	Eureka lemon	PU	16	04	7,913,464	3,410	100.0
*Citrus maxima*	Chandler pummelo	PU	18	01	2,817,860	1,283	100.0
*Citrus paradisi*	Marsh grapefruit	PU	18	01	7,090,516	3,049	100.0
*Citrus grandis*	Low acid pummelo	PU	18	01	1,305,264	577	99.9
*Citrus grandis*	Guanxi pummelo^c^	PU	18	01	2,779,065	1,302	98.5
*Citrus grandis*	Shatian pummelo^c^	PU	18	01	3,121,627	1,462	99.5
*Citrus aurantifolia*	Mexican lime	MC	108	22	3,273,200	1,479	100.0
*Citrus micrantha*	Micrantha	MC	108	22	7,656,308	3,481	99.7
*Citrus reticulata*	Mangshan mandarin^c^	MN	121	4	3,290,081	1,541	99.7
*Citrus clementina*	Clementine mandarin	MA	142	38	8,355,334	3,702	100.0
*Citrus deliciosa*	Willowleaf mandarin	MA	142	38	3,372,519	1,466	100.0
*Citrus reticulata*	Ponkan mandarin	MA	142	38	1,797,993	817	100.0
*Citrus unshiu*	Satsuma mandarin	MA	142	38	1,693,894	765	100.0
*Citrus tangerina*	Dancy mandarin	MA	142	38	3,903,551	1,779	100.0
*Citrus nobilis*	King mandarin	MA	142	38	1,651,462	750	100.0
*Citrus reticulata*	W. Murcott mandarin	MA	142	38	2,317,975	1,074	100.0
*Citrus reticulata*	Huanglingmiao mandarin^c^	MA	142	38	4,199,260	1,966	99.7
*Citrus limonia*	Rangpur lime	MA	148	40	3,847,026	1,733	100.0
*Citrus reshni*	Cleopatra mandarin	MA	149	40	8,333,902	3,592	100.0
*Citrus sunki*	Sunki mandarin	MA	149	40	1,093,257	494	100.0
*Citrus ichangesis*	Ichang papeda	PA	168	6	4,379,125	1,981	99.8
*Citrus madurensis*	Calamondin	FO	178	39	994,327	443	99.8
*Fortunella margarita*	Nagami Kumquat	FO	183	39	999,497	439	99.9
*Poncirus trifoliata*	Pomeroy	PO	216	44	12,053,896	5,359	100.0
*Citrus medica*	Buddhás Hand citron	CI	216	61	2,608,145	1,172	100.0
*Citrus medica*	Mac Veu mountain Citron	CI	216	61	3,918,552	1,766	100.0
*Citrus medica*	Humpang citron	CI	216	61	2,334,979	1,054	100.0
*Citrus medica*	Corsican citron	CI	216	61	2,096,451	955	100.0
*Microcitrus australasica*	Australian finger lime	MI	260	71	2,225,093	1,003	100.0
*Microcitrus australis*	Australian round lime	MI	293	78	1,698,579	763	99.9
*Eremocitrus glauca*	Australian desert lime	ER	362	78	1,385,587	622	100.0
*Severinia buxifolia*	Chinese box orange	SB	430	136	6,346,507	2,926	99.9

Note.—Identity of chloroplast genomes was deduced from the presence of SNVs and indels. The genome sequence of the *Citrus sinensis* chloroplast was used as the genome of reference (Bausher et al. 2006). After variant calling, SNVs and indels were manually curated. Sequencing statistics (number of mapped read, mean coverage, genome fraction with coverage over 15×) determined after excluding and inverted repeat regions and sequences homologous to nuclear DNA. Chloroplast types: PU, pummelo; MC, Micrantha; MN, mangshan; MA, mandarin; PA, papeda; FO, *Fortunella*; PO, *Poncirus*; CI, citron; MI, *Microcitrus*; ER, *Eremocitrus*; SB, *Severinia*.

^a^There were two nonconcordant positions (41051 and 78452) between the Sanger reference sequence of the *C. sinensis* chloroplast and the *C. sinensis* chloroplast genome resequenced here. The nucleotide at position 41051 in the reference sequence was not confirmed in any of the 34 genomes analyzed, whereas that at position 78452 was observed solely in the 11 mandarins clustered in group MA.

^b^Similarly, indels at positions 46206, 49034, 78458, and 83617 absent in the reference genome were observed in all 34 genomes resequenced in this work. These discrepancies that may be attributed to Sanger sequencing errors in the reference genome were not used in further comparative analyses.

^c^Those genomes were reanalyzed from original published sweet orange genome sequences (Xu et al. 2013).

### Variant Calling

Genome Analysis Toolkit (GATK) mapping followed by extensive manual curation rendered a total of 1,564 single nucleotide variants (SNVs) and 323 indels (insertions and deletions), summing up a total of 1,887 high-quality nonredundant variant positions. Manual curation and the high coverage used in this study allowed the detection of a number of discrepancies with the genome of reference that can most probably be due to Sanger sequencing errors in the original sequence (Bausher et al. 2006). Therefore, in case of discrepancy, the resequencing data were used for further comparative analyses. Furthermore, careful visual inspection also suggested that the number of indels may be slightly higher than what is reported in table 1. For example, the two pummelo chloroplast sequences that we resequenced actually contained three indels, instead of one. However, these two additional indels could not be properly resolved in the other two pummelo genomes previously reported (Xu et al. 2013) because of the absence of coverage in these regions. Interestingly, during the whole process of variant analysis it was apparent that, in spite of the haploid nature of the chloroplast genome, a number of variants were accompanied by an additional minor allele. This observation suggested that heteroplasmy may be an event more common than previously expected in the citrus chloroplast, as discussed below.

### Variant Analysis

The average SNV density in the whole chloroplast genome was 14.5 SNVs per kb. The number of changes observed for all the four nucleotides among the 1,564 nonredundant SNVs was similar (A and C, 24% and G and T, 26%). For A and T, transversions to C (64%) and G (60%), respectively, were most frequent. Conversely, for C and G, the total number of transversions (49% and 51%, respectively) and transitions (50% and 48%) were rather similar. Also 30 triallelic positions, mostly composed of G, A, and T alleles, were observed. The SNVs were rather uniformly distributed along the chloroplast genome (fig. 1). However, some regions showed an increase in the amount of SNV in comparison with the chloroplast average. These regions, spanning 4–5, 24–26, 35–38, 45–47, and 79–83 kb, might represent hotspots for genetic variation. About 50% of variant positions (781) were found in intergenic regions. The rest of variants affected to 62 genes, leaving thus a total of 51 genes unaffected. On the other hand, some genes such as *matK, rpoC2, ndhF,* and *ycf1*, displayed remarkably high SNV densities (27.2, 18.5, 19.7, and 25.2 SNVs per kb, respectively; see table 2).

**Table 2.** Genes in the Chloroplast Citrus Genome Containing SNVs and Indels.

Gene Name	Protein Name	Putative Function	Number of SNVs		Number of Indels		Indel Effect on Exon
			Intron	Exon	Intron	Exon	
*matK*	Maturase K (intron maturase)	RNA splicing	—	40	—	1	1 bp deletion
*rps16*	30S ribosomal protein S16	Translation	16	4	2	—	—
*atpF*	ATP synthase subunit b	ATP synthesis	10	2	6	—	—
*rpoC2*	RNA polymerase subunit beta	Transcription	—	78	—	1	6 bp duplication
*rpoC1*	RNA polymerase subunit gamma	Transcription	10	19	6	—	—
*ycf3*	PSI assembly protein	PSI complex assembly	13	3	3	—	—
*accD*	Acetyl-CoA carboxylase subunit beta	Acetyl coenzyme A carboxylase complex	—	24	—	2	7.18 bp duplications
*cemA*	Chloroplast envelope membrane protein	May be involved in proton extrusion	—	11	—	2	2.4 bp deletions
*petL*	Cytochrome b6-f complex subunit 6	Electron transfer between PSII and PSI	—	—	—	1	7 bp duplication
*ndhF*	NAD(P)H dehydrogenase subunit 5	PSI cyclic electron transport	—	44	—	1	6 bp duplication
*ccsA*	Cytochrome c biogenesis protein CcsA	Biogenesis of c-type cytochromes	—	19	—	1	6 bp duplication
*ycf1*	Putative membrane protein (RF1)	Essential but unknown	—	139	—	1	6 bp duplication

A total of 323 manually curated high-quality indels, ranging from 1 to 36 bp (including 21 triallelic positions), were detected. Approximately 90% of the indels were shorter than 13 bp, whereas longer indels were sporadic. Single-base indels were the most frequent ones, whereas there were fewer indels ranging from 2 to 5 bp (14%).

**Fig. 2.**
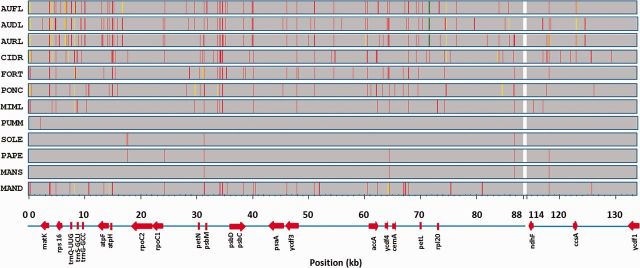
Distribution of indels along the chloroplast citrus genomes in 100-bp nonoverlapping windows. MAND, mandarins (Clementine, Willowleaf, Ponkan, Satsuma, Dancy, King, W Murcott, Huanglingmiao, Cleopatra, Sunki, and Rangpur lime); MANS, Mangshan mandarin; PAPE, Ichang papeda; SOLE, Sour orange and Eureka lemon; PUMM, pummelos and grapefruits (Chandler, low-acid, Guanxi, Shatian, and Marsh); MIML, Micrantha and Mexcian lime; PONC, Pomeroy; FORT, Calamondin and kumquat; CIDR, citrons (Buddah’s Hand, Mac Veu Montain, Humpang, and Corsican); AURL, Australian round lime; AUDL, Australian desert lime; and AUFL, Australian finger lime. The *Citrus sinensis* chloroplast genome was used as the reference genome and the two inverted repeats IRa (133–160 kb) and IRb (88–114 kb) of this genome were not included in the study. Colors represent number of indels: red: 1; yellow: 2; and green: 3. Positions of genes cited in the text are depicted as reference.

Interestingly, expansions of repeat units were virtually absent from the DNA sequences surrounding the indels, which regularly display a wide spectrum of apparently random DNA sequences. Monomeric A or T expansions were occasionally observed in a few insertions involving substitutions of T for T(A)_2,4,5,6,8_; G for G(A)_4_; C for C(A)_4_; and of C for C(T)_4_. Multibase pair expansions of repeat units were not detected while a relatively high percentage of insertions consisted of duplications of the original sequence (table 2) or even of inverted duplications of the complementary strand. Deletions of duplicated tracks were also frequently found. Insertions (59%) were more frequent than deletions (41%) and both were uniformly distributed along the *C. sinensis* chloroplast genome (fig. 2).

The analysis of the distribution of the 323 indels revealed that 296 (92%) of them were situated in intergenic regions whereas only 27 (8%) were located in genes (table 2). Seventeen out of the 27 indels were in intronic regions whereas the remaining ten indels affected the coding regions of *matK, rpoC2, aacD, cemA, petL, ndhF, ccsA,* and *ycf1* genes. Furthermore, five of these ten indels were predicted to cause frameshifts that lead to the premature termination of the encoded proteins and therefore are expected to arrest gene function. The remaining indels were duplications of 6 or 18 bp and thus resulted in insertions of additional codons. The average indel density in the chloroplast genome of citrus was 3.0 indels per kb, whereas density in coding regions was 0.5 indels per kb. There were genes, such as *atpF*, *CemA* or *rpoC1,* that displayed indel densities above the average (4.4, 2.9, and 2.2 indels per kb, respectively; see table 2).

### Phylogenetic Analysis of the Genus *Citrus*

**Fig. 3.**
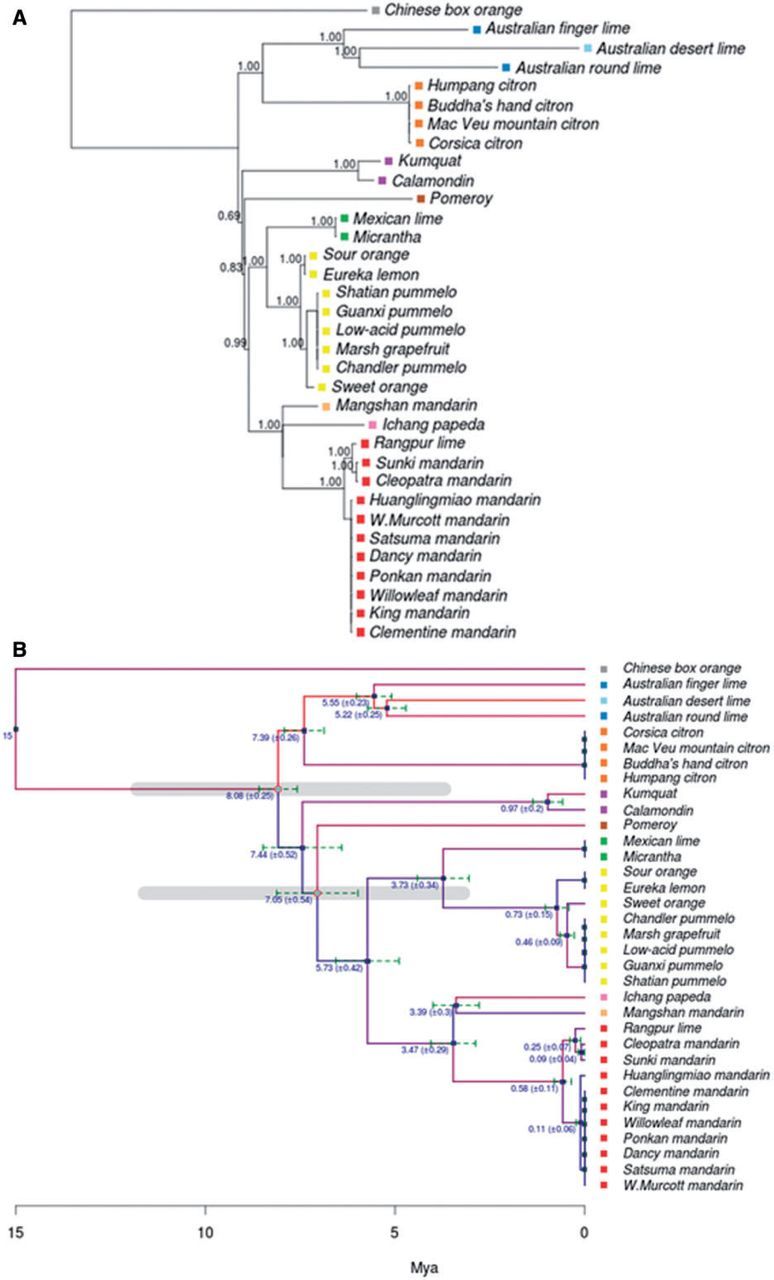
Phylogenetic tree of the genus *Citrus*. (*A*) Maximum-likelihood phylogenetic tree inferred using PhyML from the set of variant loci from 34 citrus genomes. Colors represent the main chloroplast groups (gray SB, blue MI, light blue ER, orange CI, purple FO, brown PO, green MC, yellow PU, pink PA, light orange MN, and red MA; see table 1). Bootstrap CIs for the clades are displayed in the corresponding branching points of the tree. Support values have been removed from collapsed branches. (*B*) Calibrated phylogenetic tree obtained by using correlated relaxed clock model with inferred speciation dates over a time scale in million years. The tree was calibrated using previous published estimations of citrus divergence times that date the separation between *Severinia* and the genus *Citrus* approximately 13 Ma, with a range of uncertainty from 20 to 8 Ma (Pfeil and Crisp 2008). This event was taken as the root of the tree. Other two events used for calibration were the first separation of citrus between the two main citrus clades and the *Poncirus* divergence that occurred 7.1 Ma (11.8–3.7 Ma) and 6.6 Ma (11.6–3.2 Ma), respectively (Pfeil and Crisp 2008), indicated as gray lines in the figure. 95% CIs for the speciation events, defined as 2SD, are marked as green dashed bars in the corresponding branching points. Low mutation rates are represented by blue branches, whereas high mutation rates are represented by red branches. Colored squares that precede accession names represent the main chloroplast groups.

Positions affected by SNV (a total of 1,564 sites) were concatenated and used for phylogenetic analysis. Phylogenetic trees were reconstructed as described in Materials and Methods. The results obtained by using the two different methods, MrBayes and PhyML, were almost identical, which, in addition to their corresponding internal bootstrap support, shows the reliability of the phylogeny recovered. The phylogenetic tree was rooted with *Severina buxifolia* as outgroup and showed a clustering topology in general well supported with high bootstrap values (fig. 3*A*).

The tree displayed two major citrus clades clearly separated that further diversified into different subclades. One of these clades split into a monophyletic cluster, grouping citrons, and a second cluster formed by Australasian citrus, composed of the *Microcitrus* and *Eremocitrus* genera. Intriguingly, the two *Microcitrus* accessions, *M. australasica* and *M. australis*, were not clustered together as this last species appeared more closely related to *Eremocitrus glauca* than to *M. australasica*. Regarding the other major clade, the order in which the two initial speciation events occurred was not significantly supported by the bootstrap analysis and these divisions must be therefore interpreted as the most probable succession of events. This clade was most likely first divided into a minor cluster formed exclusively by *Fortunella margarita* and *C. madurensis*, and a major cluster containing the rest of the citrus species. The subsequent most likely division separated a new monophyletic branch (the genus *Poncirus*) from the main group, which included all remaining citrus species. This main group was divided again into two branches, separating mandarins from Pummelos, two principal citrus types that are most probably directly derived from the ancestral citrus (Wu et al. 2014). The Pummelo cluster grouped several species including *C. maxima, C. grandis, C. sinensis, C. aurantium, C. paradisi,* and *C. limon* and was nested with the Micrantha group formed by *C. micrantha* and *C. aurantifolia*. Further subdivisions separated sour oranges and lemons from grapefruits, pummelos and sweet oranges, and finally these last species from the rest. In the remaining branch, the mandarin cluster was nested with a cluster including *C. ichangensis*, a citrus belonging to the subgenus Papeda, and Mangshan mandarin. The mandarin cluster was also statistically well supported and included in two separate subclusters the more “recent mandarins” (*C. reticulata, C. deliciosa, C. unshiu, C. clementine, C. tangerine*, and *C. nobilis*) and the “traditional mandarins” (*C. reshni, C. sunki,* and *C. limonia*).

### Estimation of Divergence Times in the Genus *Citrus*

The time calibration of the tree was carried out as described in Materials and Methods. Only a single best tree was found following the unconstrained (nonclock) maximum likelihood search with a −ln *L* = 9,717.403, whereas the constrained molecular clock found a tree score of −ln *L* = 11,250.99. The resulting likelihood ratio test (LRT) χ^2^ value of 3,067.174, with *N* − 2 = 32 degrees of freedom, yields a significant rejection of clocklike behavior of the data (*P* << 0.01), therefore requiring the application of relaxed-clock methods to determine node ages. The different rate classes were estimated with using a date of 16 Ma for the root. Supplementary figure S1, Supplementary Material online, shows the rates and the branches to which these are assigned. Each branch was automatically assigned to a category by using a K-means classifier. Actually, the different rates correspond to branches that have been evolving independently, after geographic isolation, as in the case of the Australian citrus, or the case of the citrons, present only in Asia (see different branches in the tree in supplementary fig. S1, Supplementary Material online). The application of the penalized likelihood (PL) (Sanderson 2003) using the two strategies (fixing the root age or applying node constrictions), as described in Materials and Methods, produced very similar results. This could be a consequence of using too wide confidence intervals (CI) for internal nodes.

All the tested root ages (from 8 to 20 Ma) show internal node ages within the expected specified constraints. The distribution of node CI length (normalized by corresponding root age) becomes stabilized from 13 to 18 Ma (see supplementary fig. S2, Supplementary Material online).

For time calibration of the phylogenetic tree inferred (fig. 3*A*) previous published estimations of citrus divergence times were used as constraints (Pfeil and Crisp 2008). According to the estimations derived from the calibrated tree, the oldest speciation event in the reported citrus phylogeny was the separation between *Severinia* and the genus *Citrus*, which occurred approximately 15 Ma. This event was assumed to define the root of the tree. Two other important events used for calibration were the first separation of citrus between the two main citrus clades and the *Poncirus* divergence that occurred, respectively, 7.1 Ma (11.8–3.7 Ma) and 6.6 Ma (11.6–3.2 Ma) (Pfeil and Crisp 2008).

Although the results rendered by the statistical model used must be taken as approximate estimations, given the low precision of the original divergence times used for the calibration, the phylogenetic tree with inferred speciation dates depicted in figure 3*B* clearly documented three main periods of extensive radiation of citrus species. According to the results, the ancestors of the current major citrus groups were generated in a succession of speciation events that started with a first radiation that occurred approximately 8.08 Ma (±0.25) separating citrons and the Australian species (*Microcitrus* and *Eremocitrus*) from the rest of citrus. The citrons and the Australian species diverged shortly thereafter (7.39 Ma ± 0.26). Although the order of the two initial splits affecting the divergence of *Fortunella* and *Poncirus* in the major citrus clade could not be accurately defined (fig. 3*A*), it took place between 7.4 and 7.0 Ma. Thus, the first radiation of citrus species took place approximately 8–7 Ma, in about than 1 My.

In a second period (which slightly overlaps with the previous one) of rapid speciation spanning approximately half million years (5.7–5.2 Ma), the different clades established in the first radiation underwent new divergent evolutionary processes.

During this second radiation, the three Australian species separated from each other (5.5–5.2 Ma), probably indicating the date in which these species started the colonization of Australia. Almost simultaneously, the cluster *Micrantha*/pummelo separated from *Papeda*/mandarins (5.73 Ma).

Later on, *Micrantha* diverged from the pummelo cluster (3.73 Ma ± 0.34) and the papeda/Mangshan/mandarin cluster divided into three branches approximately 3.4 Ma.

Finally, a more recent forth radiation period (0.9–0.1 Ma) produced, through new speciation events, the citrus genus as we know it at present. During this recent period, the *Fortunella* cluster split into two different species, calamodin and Kumquat, the pummelo clade generated the ancestors of sour and sweet oranges (about 400,000 years ago), lemons and grapefruits, and the radiation of mandarins produced two different subclusters. It is worth mentioning that, although the evolution of these species could have started several hundred thousand years ago, the estimation of recent speciation dates for current edible species, as mandarins and oranges, may be influenced by domestication or random crosses during historic times.

### Heteroplasmy Events in the Citrus Chloroplast Genome

The analysis of SNVs and indels in the citrus chloroplast genomes revealed a pervasive presence of accompanying alleles at lower frequencies. This observation suggests that heteroplasmy, an event generally attributed to mutations or biparental inheritance, was more frequent than previously expected in the *Citrus* genus. In the case of biparental inheritance, the genomic identity of the heteroplasmic positions in the paternal parent species confirms the heteroplasmic sites in the progeny.

In order to detect heteroplasmic positions, we performed a study of the genomic structure of the 1,564 nonredundant positions corresponding to the SNV observed in all citrus chloroplast genomes. A position was defined as heteroplasmic when the minor allele was present in at least 5 out of a minimum of 1,000 reads. Sequences with heteroplasmic positions were manually curated.

**Fig. 4.**
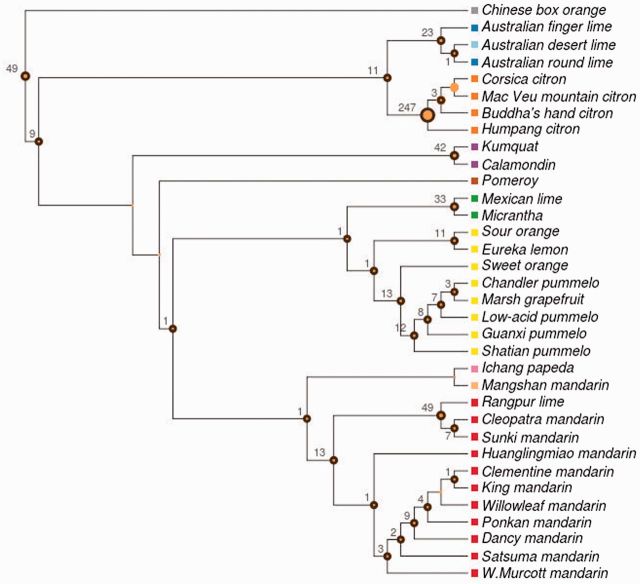
Ancestral inference of heteroplasmic events per node. Node size is proportional to the amount of heteroplasmic events occurred on each corresponding node. Colors represent the main chloroplast groups as indicated for figure 3. Circles with black border indicate hypothetical origins of heteroplasmic events.

Table 3 shows the levels of heteroplasmy among the citrus clusters depicted in the phylogenetic tree presented in figure 3*A*. The data showed that the cluster of *C. medica* was the most homogeneous group regarding heteroplasmy, as citrons exhibited a very high percentage of shared heteroplasmic positions (93%) and the highest number of total positions per genome (268). Other clusters also showed high percentages of heteroplasmic shared sites, such as the *Micrantha*–mexican lime (76%). The Calamondin–Kumquat cluster, the Pummelos and three mandarins included, displayed shared single heteroplasmic positions for half of the total sites, although the number of total heteroplasmic alleles in these clusters was somehow lower. The cluster formed by sour orange and lemon exhibits the highest ratio of shared positions (95% of 22 sites). Regarding the rest of the branches, the low percentage of heteroplasmic positions shared found in the Papeda–Mangshan cluster (10%) is consistent with the genetic distance between these two species (fig. 3*A*). In contrast, the presence of identical variations can be easily recognized in the mandarin A cluster (31%), composed of hybrid origin mandarins, and in the three Australian species (22%). Overall, these observations reinforced and provided an independent support to the main clusters and the topology of the phylogenetic relationships based on SNVs presented above (fig. 3*A*). Figure 4 shows the inference of ancestral heteroplasmy and the inference of the ancestral origin of the heteroplasmy shared by the different species.

**Table 3.** Heteroplasmy Levels in Citrus Clusters Calculated as the Ratio between the Shared and the Total Heteroplasmic Positions in Each Cluster.

Citrus Clusters	Mandarin		Papeda Mangshan	Pummelo	Lemon Sour Orange	Micrantha Mexican Lime	Calamondin Kumquat	Citrons	Australian Species
	A	B							
Species, number	7	3	2	5	2	2	2	4	3
Single heteroplasmic positions									
Total^a^	55	100	41	60	22	46	90	268	153
Shared^b^	17	53	04	36	21	35	46	250	34
Percentage	31	53	10	50	95	76	51	93	22

Note.—Shared heteroplasmic positions were searched among the 1,564 nonredundant positions corresponding to the SNV set, in all citrus chloroplast genomes. A certain position was defined as heteroplasmic when the minor allele was present in at least 5 out of a minimum of 1,000 reads. Clusters correspond to those depicted in the phylogenetic tree presented in figure 3. The mandarin A cluster includes all species of mandarins known (or assumed) to have a hybrid origin, except Huanglingmiao mandarin. The mandarin B cluster includes the species of uncertain origin. The pummelo cluster also includes a grapefruit species.

^a^Total heteroplasmic positions in a cluster. For comparison between four or more species, the average number of single heteroplasmic positions among these species was used; otherwise, the lower number found in a species was utilized.

^b^Shared heteroplasmic positions in a cluster. Shared positions in all species of a specific cluster are reported, except in the mandarin cluster A (positions shared by at least six species) and in the pummelo cluster (positions shared by at least four species).

These results indicated that the most likely origin of heteroplasmy in citrus chloroplasts is biparental inheritance in intergeneric or interspecific crosses (Moreira et al. 2002). Thus, heteroplasmic positions can be used to infer the origin of the minor frequency allele through comparative analyses of the genomes sequenced. The presence of paternal parent chloroplasts was explored in several genotypes known (or assumed) to be hybrids, such as Rangpur lime (*C. limonia*), mexican lime (*C. aurantifolia*), lemon (*C. limon*), sweet orange (*C. sinensis*), and Pomeroy (*Poncirus trifoliata*). Table 4 shows the results of the comparison of shared heteroplasmic positions between any hybrid and its putative paternal parents. According to the ratio of heteroplasmic compatible positions a citron was the most likely paternal parent of *C. limonia* (ratio of compatibility 0.97–0.94), of *C. aurantifolia* (0.96–0.91), and of *C. limon* (0.93–0.88). The results also confirmed that sweet orange resulted from a hybridization of Pummelo with mandarin as paternal parent, as the compatibility among the heteroplasmic positions of sweet orange and the corresponding sites in each of the 11 mandarins tested was always very high (0.92–0.82). Interestingly, the analyses also suggested that the putative paternal parent of *Poncirus* was a noncitrus genotype, given that *Severinia* showed the highest compatibility ratio (0.82) although low and not very different from the fundamental citrus groups (citrons, pummelos, mandarins and Australian limes, with indexes ranging from 0.81 to 0.78). This observation opens the possibility of an intergeneric hybridization as the potential origin of *Poncirus*.

**Table 4.** Heteroplasmy Analyses of Biparental Inheritance of Citrus Hybrids.

Hybrid	Maternal Parent	Paternal Parent	Number of SHPs		
			Incompatible	Compatible	Ratio
*C. limonia*	Mandarin					
		Citrons (4)	7–16	263–254	0.97–0.94
		Australian limes (3)	106–122	164–148	0.61–0.55
		Chinese box orange (1)	137	133	0.49
		*Poncirus trifoliata* (1)	147	123	0.46
		Fortunellas (2)	156–159	114–11	0.42–0.41
*C. aurantifolia*	*Micrantha*				
		Citrons (4)	12–23	256–245	0.96–0.91
		Australian limes (3)	126–140	118–104	0.48–0.43
		Chinese box orange (1)	147	121	0.45
		*Poncirus trifoliata* (1)	159	109	0.41
*C. limon*	Sour orange				
		Citrons (4)	18–30	226–214	0.93–0.88
		Australian limes (3)	126–140	118–104	0.48–0.43
		Chinese box orange (1)	145	99	0.41
		*Poncirus trifoliata* (1)	162	82	0.34
		Orange (1)	168	76	0.31
*C. sinensis*	Pummelo				
		Mandarins (10)	14–30	156–140	0.92–0.82
		Papeda (1)	79	91	0.54
		Mansghan (1)	80	90	0.53
		Citrons (4)	97–93	77–73	0.45
		Australian limes (2)	97	73	0.43
*P trifoliata*	*Poncirus*				
		Chinese box orange (1)	17	79	0.82
		Citrons (4)	18–21	78–75	0.81–0.78
		Pummelo (1)	23	73	0.76
		Mandarin (1)	24	72	0.75
		Australian lime (1)	24	72	0.75

Note.—Biparental inheritance was determined as the compatibility ratio of single heteroplasmic positions (SHPs) between each hybrid and the putative paternal parent. SHPs were searched in the 1,564 nonredundant positions corresponding to the SNV set. A certain position was defined as SHP when the minor allele was present in at least 5 out of a minimum of 1,000 reads. The analyses were performed against the whole collection of sequenced accessions although the table shows the top five hits for putative paternal parents containing more than 70 heteroplasmic compatible positions. Numbers between parentheses after the citrus groups indicate number of species involved in the estimation of the values.

As previously mentioned, the coverage is very high (over 2,000×; see table 1) and the pattern of heritage 100% coherent with the phylogeny, which clearly indicates that the results obtained were highly reliable. However, we have carried out an extra validation by a different technology to be sure that the observations were not due to any unexpected technological bias. Sanger sequencing was carried out in two heteroplasmic sites in the trio mandarin (maternal parent), citron (paternal parent), and the hybrid *C. limonia* (positions 21826 and 20848, see supplementary fig. S3, Supplementary Material online) and also in the trio Micrantha (maternal parent), citron (paternal parent), and the hybrid *C. aurantifolia* (position 69792, see supplementary fig. S3, Supplementary Material online). The Sanger sequencing results clearly reveal the minority but clear presence of the paternal allele in the three cases analyzed (supplementary fig. S3, Supplementary Material online). Paradoxically, Sanger sequencing is by far less sensitive than next generation sequencing at the coverage used here (over 2,000×; see table 1) and therefore, only heteroplasmic positions with a relatively high proportion of paternal allele (over 10%) can be confirmed with this technique.

The analyses of heteroplasmic alleles in the chloroplast genomes revealed that heteroplasmy was a relatively frequent event in the *Citrus* genus.

### Structural Variants in the Citrus Chloroplast Genome

**Fig. 5.**
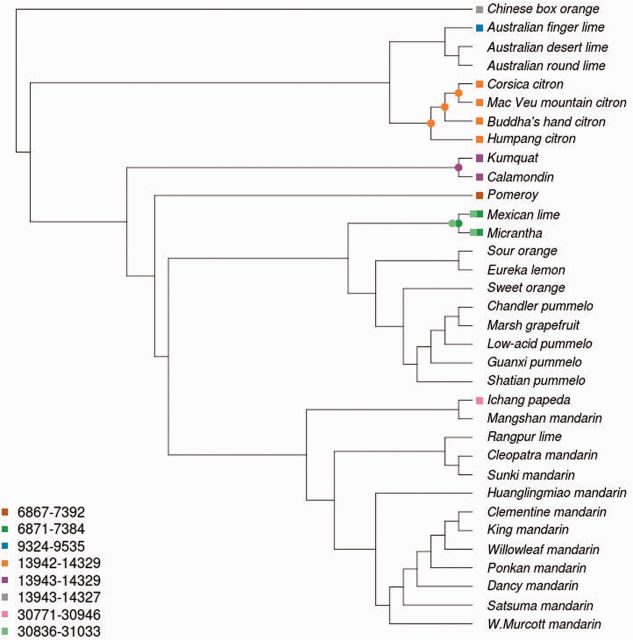
Distribution and evolution of structural variants along the citrus phylogeny. Eight structural variants observed in the different species are represented as squares of different color. The figure legend represents the genomic coordinates of the different SVs (some of them overlap due to the small portion of the chloroplast genome that can be lost without affecting functionality). The inferred evolutionary origin of the different SVs along the evolutionary tree of the *Citrus* genus is represented with a circle of the same color than the corresponding SV.

Chloroplast genomes are small, compact and highly conserved and consequently not much SV was initially expected. A careful analysis, carried out under very stringent criteria (see Materials and Methods), revealed the presence of at least eight highly confident SV events in the genus *Citrus* (fig. 5). All the SVs reported here were deletions located in intergenic regions and most of them generated recognizable heteroplasmic events. Seven of them were overlapping deletions in three different positions with their breakpoints and junctions occurring in homologous genomic regions. This suggested that deletions were most probably generated through crossovers between homologous regions. Moreover, their occurrence in several citrus species allowed the study of their fixation along evolution.

**Fig. 6.**
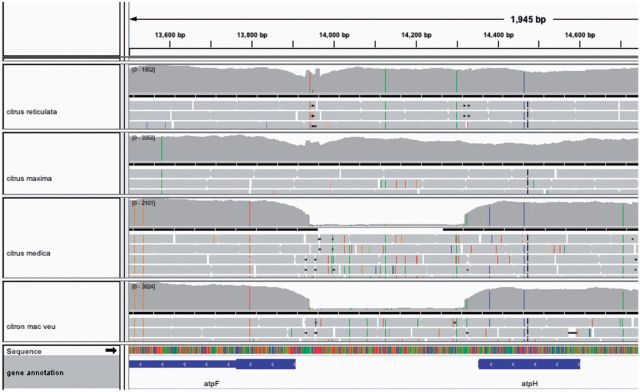
Observed coverage of mapped reads supporting the existence of a large deletion. IGV screenshot of the variability and coverage observed in four citrus sequence samples. Upper panel represents the genomic coordinates. There are four panels corresponding to the different citrus sequences. Each of these panels is subdivided into different racks. The upper track describes the density of read mapping. Then, there are three or four tracks that represent the three or four rows of mapping sequences. Thin dashes across the reads represent positions that change in the read with respect to the *Citrus sinensis* chloroplast reference genome. The two panels in the bottom of the figure represent the lineal DNA sequence with all the variants found and the genes and other genomic annotations. The deletion is located in a narrow genomic region between the atpF and atpH genes. In the example, two species, *Citrus maxima* and *Citus reticulata* have the region whereas *Citrus medica* and *Citron mac veu* display a clear deletion.

**Fig. 7.**
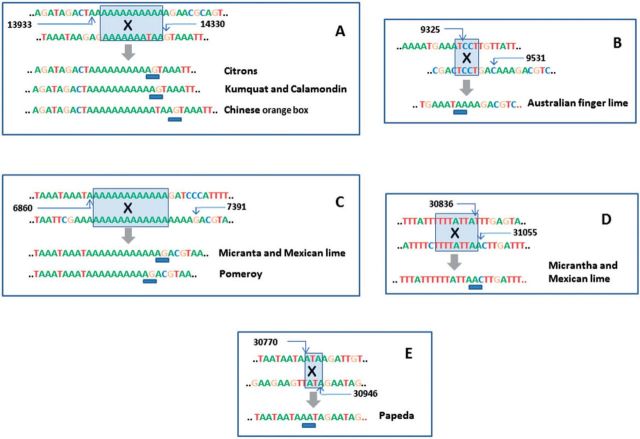
Sequences of breakpoint flanking regions of various deletions in chloroplast genomes of citrus. In each panel, the two first sequences that contain reference positions, correspond to the two separate regions involved in the deletions while the resulting rearrangement are indicated below the wide arrow. The shade boxes denote homologous stretches in the original sequences; the X indicates putative crossovers between the homologous sequences and the blue bars show the exact junction. (*A*) All citrons share a deletion spanning positions 13943–14330 that overlaps with an independent deletion spanning positions 13944–14330 in Kumquat and Calmondin and with that of Chinese orange box at positions 13946–14330. (*B*) Australian finger lime contains a deletion spanning positions 9325–9531. (*C–E*) Micrantha and Mexican lime share two deletions; one of them spanning positions 6871–7391 overlaps with an independent deletion spanning positions 6869–7391 in Pomeroy. The other deletion, spanning positions 30836–31055 partially overlaps the deletion at positions 30770–30946 of Papeda.

At least three independent deletions that were essentially identical (except for differences of one or two bases in the breakpoints) were observed in different citrus genotypes in the same narrow stretch genomic region located between the *atpF* and *atpH* genes (fig. 1 and see detail in fig. 6). The two stretches flanking the coding regions of these two genes had a similar structure: A 9–15 poly(dA) homopolymer separated by 25 bp of the corresponding coding regions (fig. 7*A*). These three deletions affected the fragment located between both homopolymer tracks. However, they also showed heteroplasmic genomic structures, that is, the reference allele was observed at low frequency. The deletion spanning positions 13943–14330 was exclusively detected in all chloroplast genomes of citrons, suggesting that it occurred after their split from the Australian species. Another overlapping although independent deletion (spanning positions 13944–14330) in this region affected exclusively Kumquat and Calamondin, indicating that the deletion was generated in the lineage leading to these two species. There was still an additional overlapping deletion (spanning positions 13946–14330) identified in the *Severinia* chloroplast genome, the outgroup used in the phylogenetic study. This observation supports both the independent origin of the three overlapping deletions and the recurrent predisposition of this genomic stretch to undergo deletions.

The Australian finger lime contained a deletion located between two tRNAs genes, *trnS-GCU* and *trnG-GCC*, spanning positions 9325–9531. This deletion was not present in the other two Australian species, suggesting that the event occurred after the separation of these species. The homologous region that probably underwent crossover in the generation of this deletion was the TCCT tetranucleotide (fig. 7*B*).

The two species included in the Micrantha/Mexican lime cluster showed two additional deletions also located in intergenic regions, spanning positions 6871–7391 between genes *rps16* and *trnQ-UUG* (fig. 7*C*), and 30836–31055, between genes *petN* and *psbM* (fig. 7*D*). The deletions were identical in both species, supporting that Micrantha and Mexican lime shared the same chloroplast genome as expected in a mother–child relationship. However, very similar, overlapping but not identical deletions were, respectively, observed in Pomeroy and Papeda (fig. 7*C*), two species not directly related according to the phylogenetic tree depicted in figure 3*A*. One of the deletions was probably generated through crossover between homopolymers as the breakpoints of this deletion for *Micrantha*, Mexican lime, and Pomeroy were located in the same 13 and 19 long poly(dA) track. Although Micrantha and Mexican lime contained exactly the same rearrangement, Pomeroy exhibited a 2-bp longer deletion (spanning positions 6869–7391). In contrast, the homologous tracks involved in the other deletion were different in Micrantha/Mexican lime (TTTTATTA) and in Papeda (ATA), generating in this way different but still overlapping deletions (30836–31055, Micrantha/Mexian lime; 30770–30946, Papeda; fig. 7*D* and *E*).

Overall, these observations provide support to the phylogenetic relationships depicted in figure 3*A* indicating that the members of each group, that is, citrons, Kumquat/Calamondin and Micrantha/Mexican lime, they all shared the same SVs in the chloroplast genome. The independent origin of the overlapping deletions also indicates that these positions show a relatively high predisposition to generate rearrangements.

### Selective Pressures in the Evolution of the *Citrus* Genus

**Fig. 8.**
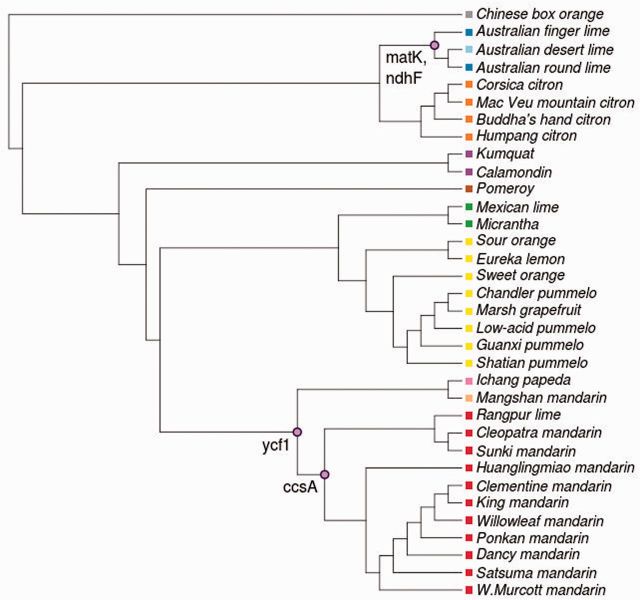
Events of positive selection in genes located in the corresponding lineage of the evolutionary tree. Colors represent the main chloroplast groups (gray SB, blue MI, light blue ER, orange CI, purple FO, brown PO, green MC, yellow PU, pink PA, light orange MN, and red MA). Circles represent branches of the tree where genes were significantly under positive selection. The names of the genes are represented near the circles.

Finally, the analyses of selective pressures along the phylogenetic tree (see Materials and Methods) unambiguously detected four genes (*matK, ndhF, ycf1*, and *ccsA*) under positive selection (fig. 8). Interestingly, these four genes were previously found to present SNV and indel densities clearly above the average affecting specifically exonic regions (table 2). Genes *matK* and *ndhF* were under positive pressure exclusively in the Australian clade. Gene *ycf1* was positively selected in the *Papeda/Mangshan/mandarin* cluster, whereas *ccsA* selective process seems to be linked to the emergence of mandarins (fig. 8).

## Discussion

The sequencing of 34 complete chloroplast genomes representative of the main citrus species (table 1) has certainly added new and valuable information to solve important controversies on the evolution of the genus (Scora 1975; Morton et al. 2003; Pfeil and Crisp 2008; Bayer et al. 2009; Li et al. 2010; Garcia-Lor et al. 2013; Penjor et al. 2013). The genomes sequenced allowed us to derive a highly reliable phylogenetic tree based on a set of 1,564 high-quality SNVs, rooted with *S. buxifolia* as outgroup (fig. 3*A*). Additional studies of indels (see fig. 2), large structural variants (SVs; see fig. 5), and heteroplasmic positions (tables 3 and 4 and fig. 4) rendered patterns of distribution across species compatible with the topology of the phylogenetic tree, providing an independent support to our proposal.

There is a general agreement in the monophyletic origin of all citrus despite the topologies reported varies considerably. Most studies reported a superior clade including at least five related genera: *Citrus, Microcitrus, Eremocitrus. Fortunella*, and *Poncirus*. On the other hand, the genus *Citrus* has traditionally been divided into two subgenera: *Citrus* and *Papeda* (Swingle and Reece 1967). The subgenus *Citrus* is invariably divided into three major groups that are coincident with the classification of the three main true citrus species (Scora 1975; Barrett and Rhodes 1976): Citrons, pummelos, and mandarins. However, the grouping of the three clusters is rather controversial because the different studies used different DNA sequences, either from the chloroplast (Green et al. 1986; Nicolosi et al. 2000; Araujo et al. 2003; Li et al. 2007, 2010; Bayer et al. 2009; Lu et al. 2011; Penjor et al. 2013) or from the nucleus (Garcia-Lor et al. 2013), for their definition.

According to our results, the citron cluster separated from a second cluster that diverged thereafter in pummelos and mandarins (fig. 3*A*). Furthermore, the study of distribution of both indels (table 1 and fig. 2) and heteroplasmic positions (table 3) indicated that citrons are the most distantly related species among these three citrus groups. Our results also support that the citron cluster belonging to the subgenus *Citrus* was nested with the Australasian citrus, composed of the genera *Microcitrus* and *Eremocitrus*. Interestingly, the grouping of these three species showed that the two *Microcitrus* were not clustered together, as previously proposed (Li et al. 2007; Bayer et al. 2009; Lu et al. 2011; Garcia-Lor et al. 2013). Estimations of divergence times presented figure 3*B* suggested that this first event of speciation that separated the citrons/Australian species clade from the rest of citrus, occurred approximately 8.08 Ma (±0.25). Citrons and Australian species diverged shortly thereafter (7.39 Ma ± 0.26). In this cluster, two different large deletions, one of them affecting all citrons and the other one affecting the Australian finger lime, were detected, indicating that these events likely occurred after the separation of these species (fig. 5).

The *Fortunella* clade split into two different species, calamodin and Kumquat (0.97 Ma ± 0.2). This observation is supported by a high bootstrap in the phylogenetic tree (fig. 3*A*), the ratio of shared heteroplasmic positions (table 3), and the occurrence of a deletion exclusively shared by the two species (fig. 5).

The genus *Poncirus* formed a monophyletic lineage within the *Citrus* group, as generally reported in analyses based on chloroplast sequences (Araujo et al. 2003; Morton et al. 2003; Li et al. 2007; Bayer et al. 2009; Penjor et al. 2010; Lu et al. 2011). However, *Poncirus* shows remarkable anatomical and physiological differences with respect to citrus and analyses of nuclear molecular markers indicated that both genera may be rather distant (Nicolosi et al. 2000; Barkley et al. 2006; Garcia-Lor et al. 2013). The observed distribution of indel (fig. 2) suggested that *Poncirus* might actually be more distantly related to citrus than *Fortunella*. Moreover, phylogenetic data clearly indicated that the maternal parent of *Poncirus* is a citrus species (fig. 3*A*) whereas the biparental heteroplasmic analyses presented in table 4 suggested that the paternal parent of *Poncirus* maybe a distantly related citrus or even a noncitrus species. *Poncirus* also contained a large deletion not shared by any other genotype (fig. 5).

The pummelo cluster (generated 3.75 Ma ± 0.34) has a strong statistical support (fig. 3*A*), with additional confirmation from the distributions of indel and heteroplasmic position (tables 1 and 3). This clade grouped species such as *C. aurantium, C. limon, C. sinensis, C. maxima,* and *C. paradisi*.

Again, reports regarding the phylogenic relationships within the subgenus *Papeda* are conflicting (Nicolosi et al. 2000; Asadi Abkenar et al. 2004; Deng et al. 2007; Bayer et al. 2009; Li et al. 2010; Penjor et al. 2010, 2013; Froelicher et al. 2011; Garcia-Lor et al. 2013). Our results significantly support that Micrantha and Mexican lime were nested with the pummelo cluster, whereas in the other clade *C ichangensis* and Mangshan mandarin diverged (3.39 Ma ± 0.3) from the mandarin cluster (fig. 3*A* and *B*). Furthermore, both Micrantha and Mexican lime contained two different large deletions that were absent in Papeda, which in contrast exhibited an additional deletion (fig. 5). These results suggested that Micrantha and *C. ichangensis* are not related species and apparently invalidated the traditional subdivision between the subgenera *Citrus* and *Papeda* (Swingle and Reece 1967).

According to chronological estimations in figure 3*B*, the radiation of mandarins occurred very recently (0.58 Ma ± 0.11) and produced two different subclusters that separated “traditional mandarins” (*C. reshni*, *C. sunki*, etc.) from mandarins that are believed to be more modern mandarins (*C. reticulata, C. deliciosa, C. unshiu, C. clementine*, etc.) This clustering is also statistically well supported, and additional support is provided by distributions of indels and heteroplasmic positions which are coherent with the topology (tables 1 and 3). This observation is relevant because most of the previous studies based on chloroplast and mitochondrial DNA (Yamamoto et al. 1993; Nicolosi et al. 2000; Urasaki et al. 2005; Deng et al. 2007; Li et al. 2007; Froelicher et al. 2011; Penjor et al. 2013) have reported a single mandarin cluster with no additional subclustering (with a few controversial exceptions [Li et al. 2010; Lu et al. 2011; Garcia-Lor et al. 2013]). Our results also cluster the Mangshan mandarin outside of the two “actual” mandarin subclusters. This “mandarin” showed an abnormally low number of indels (table 1) and according to the distribution of heteroplasmic positions it was not closely related to the Papeda species (table 3). Moreover, a recent comparative genome analysis (Wu et al. 2014) showed that Mangshan mandarin displayed substantial sequence divergence from *C. reticulata*, suggesting that Mangshan represents a distinct taxon.

Estimations of the age of the genus *Citrus* in the literature are very scarce, controversial and contain a high degree of uncertainty, consequently, caution must be taken with the estimations rendered by our analyses. In spite of the associated uncertainty, the calibrated tree presented in figure 3*B* clearly depicted an evolutionary scenario with three main periods of speciation, which can be considered the first attempt to date the evolutionary history of the members of the genus *Citrus*.

As the chloroplast phylogeny represents exclusively the evolution of the maternal citrus lineage, the maternal parent species and the hybrid genotype will cluster together. In the genomes analyzed here, a number of genotypes are known (or assumed) to be hybrids and consequently showed this clustering pattern. Several reports (Nicolosi et al. 2000; Penjor et al. 2013), not exempt from some controversy (Deng et al. 2007; Penjor et al. 2010), suggested that *C. micrantha* was the maternal ancestor of *C. aurantifolia.* Furthermore, the recent sequencing of the citrus nuclear genomes (Wu et al. 2014) indicated that sour (*C. aurantium*) and sweet oranges (*C. sinensis*) were basically interspecific hybrids of *C. maxima* and *C reticulata*. It is also known that *C. paradisi* is an offspring of *C. maxima* *× C. sinensis.* Similarly, several “modern mandarins” including Clementine (Wu et al. 2014), King, and W. Murcott are assumed to be sweet orange mandarin hybrids or descendants of admixed hybrids. All these genotypes plus the putative hybrids from *C. medica,* lemon (*C. limón; C. aurantium* *× C. medica*) and Rangpur lime (*C. limonia; C. reticulata* *× C. medica*) (Barrett and Rhodes 1976; Nicolosi et al. 2000; Barkley et al. 2006; Li et al. 2010; Garcia-Lor et al. 2012, 2013; Ollitrault et al. 2012) were used in our study (table 1). Our results confirmed all these maternal–child relationships through several lines of evidence. First, the topology of the chloroplast phylogeny was fully compatible with this view suggesting that sour orange/lemon and sweet orange/pummelo/grapefruit, all of them having pummelo as the maternal parent. The phylogenetic tree also grouped Micrantha and Mexican lime on one hand, and Rangpur lime and mandarins on the other hand, as two separated branches, indicating that the Mexican lime certainly has a Micrantha type chloroplast whereas the maternal parent of Rangpur lime was a mandarin. Furthermore, the indel (table 1) and the heteroplasmic site (table 3) distributions also agreed with the hybrid nature of these genotypes. Additionally, the biparental heteroplasmic test presented in table 4 established with high reliability that Rangpur lime (*C. limonia*) was a *C. reticulata* *× C. medica* hybrid, Mexican lime (*C. aurantifolia*) was a *C. micrantha* *× C. medica* offspring, and lemon (*C. limon*) resulted from a *C. aurantium* *× C. medica* cross and also confirmed that *C. sinensis* had a mandarin as paternal parent.

In fact, a remarkable level of heteroplasmy, represented by different genomic configurations (including single heteroplasmic positions, heteroplasmic indel alleles, and even heteroplasmic stretches present in larger deletions), was observed in the analyzed species. The analyses of these heteroplasmic occurrences revealed that the set of alleles present at minor frequency often coincided with the chloroplast genome sequence of other citrus, suggesting that events of chloroplast hybridization, that is, biparental inheritance, have occurred. In the case of interspecific or intergeneric hybrids, some readjustment or mismatches in the reproductive process might generate unusual inheritance patterns of cytoplasmic genomes. An example of this is the partially biparental mitochondrion inheritance detected in citrus–Poncirus crosses (Moreira et al. 2002). Therefore, this scenario is compatible with the intrusion of a small amount of external chloroplasts into the maternal cell rather than with the arising and fixation of multiple mutations. In biparental inheritance, the genomic identity of the heteroplasmic positions in the paternal parent pinpointed directly to the genomic structure of the heteroplamic sites in the progeny. Consequently, heteroplasmic alleles in general were not distributed randomly but rather were shared by species in the same clade (table 3). These results supported the reliability of the observations made and proved that minor frequency alleles from heterogenic chloroplasts can successfully be fixed in the descendants.

**Fig. 9.**
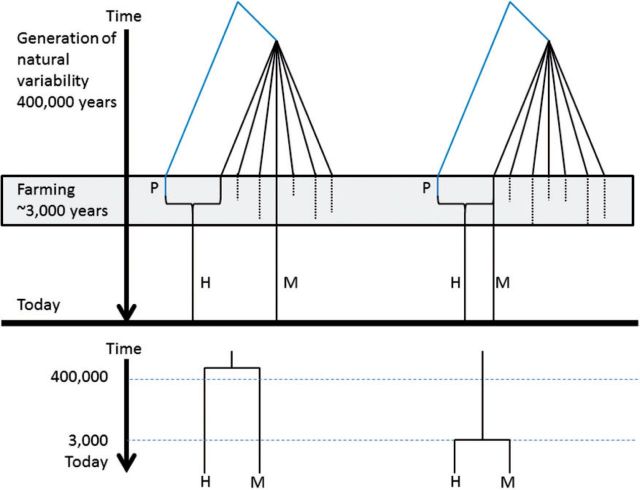
Two possible scenarios for the generation of hybrids. On the left part of the figure, the variability of the species is generated time ago (400,000 years) one of the varieties is cultivated (M, which is the maternal parent species). A different species (P) is crossed as paternal parent with another variety, whose chloroplast genome is (except for the heteroplasmy traces) lost. Both variants are cultivated systematically 3,000 years ago and most of the natural variability of the species disappears. The phylogeny recovered will reflect the moment in which the intraspecific variability was generated 400,000 years ago. A completely different scenario is shown in the right part of the figure. In this case, the variety chosen for the cross is the one which is cultivated. Then, the resultant phylogeny will reflect the moment of the cross, about 3,000 years ago.

Interestingly, we have observed two scenarios among these crosses. In most of the cases (e.g., Eureka limon—*C. limon*—and sour orange or Rangpur lime—*C. limonia*—and mandarin), the hybrid is very close to the maternal parent and the estimated date is compatible with the period of domestication (about 3,000 years ago), as expected. However, there are cases as the sweet orange, which seems to have diverged from its maternal parent, the pummelo, about 460,000 years ago. Nevertheless, the sweet orange is believed to have been originated by a natural cross between 2,000 and 3,000 years ago (Xu et al. 2013). Probably this is because the variety that was crossed and the variety that was cultivated were different and therefore the phylogeny is reflecting the moment in which the natural variability of the species was generated (fig. 9 illustrates this idea). Alternatively, the culture of sweet orange could have introduced an enormous amount of variability, but this explanation is less likely, given that in other cultivated species this artificial increase in branch lengths is not observed.

In addition to heteroplasmy, SNVs, and small indels, chloroplast genomes may undergo major structural mutational processes, such as complex recombination events, larger rearrangements, duplications, deletions, and even transfer events (Timmis et al. 2004; Raubeson and Jansen 2005). As large SVs are not expected to be frequent in a compact and conserved genome like the chloroplast (Raubeson and Jansen 2005), a stringent analysis was performed to detect them. All SVs reported here were deletions and some of them were detected in species with a common ancestor (fig. 5) providing additional support to the phylogenetic relationships derived here (fig. 3*A*). Furthermore, all the eight deletions found were located in intergenic regions (fig. 1), seven of them were overlapping deletions in three different positions (fig. 5). These observations are not surprising as most of the DNA sequence of the chloroplast genome appears to be essential for normal functioning of cellular machinery.

In addition to these four regions, we also found DNA stretches with higher SNV and indel variability. These regions spanned 2–4 kb (see SNV tracks in fig. 1) and a few more kilobases in the case of indels (fig. 2) and basically show no overlap. In contrast, the four large deletions were located in three regions of indel accumulation (fig. 1), suggesting that these fragments certainly are more prone to generate structural variability. Several coding regions accumulated a higher number of variants compared with the average regions of the genome. Six specific genes (*matK, rpoC2, accD, ndhF, ccsA,* and *ycf1*) accumulated simultaneously a high number of SNVs (≥19) and at least one indel in the coding region (table 2). This observation suggested that these genes may function as general hotspots of natural genetic variation in citrus, reflecting the occurrence of several alleles that are maintained under selective pressure because they provide some advantage.

The analysis of positive selection along the *Citrus* phylogeny unambiguously detected positive selection pressures in four genes: *matK, ndhF, ycf1,* and *ccsA* (fig. 8). Two of these genes, *matK* and *ndhF*, were under positive pressure exclusively in the Australian clade and have been explicitly reported to show very elevated substitution rates in other plants. The *matK* gene encodes a maturase involved in splicing type II introns from RNA transcripts required for normal chloroplast function. This gene in particular has been profusely used in several species, including *Citrus* (Penjor et al. 2013), as a valuable gene for DNA barcoding in phylogenetic and evolutionary studies. In a survey comparing 70 plant groups, positive selection of this gene was reported in about half of these groups, suggesting that the contrasting ecological conditions between the different plant groups may have imposed distinct selective pressures on the *matK* gene (Hao et al. 2010). The other gene under positive pressure in the Australian species, *ndhF*, encodes subunit 5 of the chloroplast NAD (P) H dehydrogenase (NDH) complex involved in photosystem I (PSI) cyclic electron transport (Peng et al. 2010). In addition to the contribution of chloroplast NDH to proton gradient formation, the driving force guiding NDH evolution appears to be related to its involvement in alleviating oxidative stress in chloroplasts. Chloroplast NDH monomers, for instance, are sensitive to high-light stress and therefore, might have changed developing novel functions for stress acclimation resistance (Peng et al. 2010).

During the formation of the clade that leads to Papeda and mandarins a strong positive selection also affected gene *ycf1*, encoding for a putative membrane protein that appears to be essential for cell survival in tobacco (Drescher et al. 2000). This gene, however, has been shown to be subjected to major rearrangements. Thus, in *Arbutus unedo*, the *ycf1* gene remains nonfunctional as pseudogene (Martinez-Alberola et al. 2013) and in other plants it has been deleted whereas in sesame it has been transferred to the nucleus (Zhang et al. 2013). The fourth gene under positive selection, *ccsA*, was detected in the mandarin clade. *CcsA* encodes for protein *CcsA*, a component of the cytochrome c synthase complex of the membrane-bound System II machinery for cytochrome c biogenesis. In Saxifragales, for instance, it has been reported that *matK* and *ccsA* genes have evolved more rapidly than other gene groups (Dong et al. 2013).

Although understanding how genes can accommodate elevated rates of nucleotide substitutions and yet maintain normal functionality it is still an open question, it is interesting to note that in citrus two faster-evolving genes, *matk* and *ndhF* showed signatures for positive pressure in a citrus clade, the Australian species, that presumably underwent trough major adaptive processes during acclimation to a dry environment.

## Conclusions

This work reports a comparative analysis of the chloroplast genomes of 34 citrus genotypes and presents a comprehensive study of their phylogenetic relationships and divergence time estimations. The analyses identified the genomic SNVs, indels, heteroplasmic positions, structural variants, and fast evolving genes occurring in chloroplast genomes of the genus *Citrus*. The results indicated that this genus is composed of three main clades, the citron/Australian species, the pummelo/Micrantha, and the papeda/mandarins. The *Citrus* ancestors were probably generated in a succession of speciation events occurring between 7.5 and 6.3 Ma, followed by a second radiation (5.0–3.7 Ma) that separated citrons from Australian species, Micrantha from *Pummelos,* and *Papedas* from mandarins. Further radiation of *Fortunella*, sour and sweet oranges, lemons, and mandarins took place later (1.5–0.2 Ma). As recently described for other organelles, such as human mitochondria (Hodgkinson et al. 2014), chloroplast genomes of citrus contained a remarkable level of heteroplasmy predominantly due to biparental inheritance. On a finer scale, we also identified six genes that may be general hotspots of natural genetic variation in citrus whereas positive selection was unambiguously detected in four of these genes*, matK, ndhF, ycf1,* and *ccsA*.

## Materials and Methods

### DNA Extraction and Plant Material

Total genomic DNA for genome sequencing was extracted exclusively from fresh young leaves basically as described in Terol et al. (2015). Leaves were ground, buffer was added, and samples were homogenized and filtered through layers of Miracloth. Extracts were centrifuged twice, the pellet resuspended in floating buffer and centrifuged again. DNA was recovered by pipetting, homogenized, resuspended in nuclear buffer, and centrifuged. The supernatant was discarded, RNase and protein Kinase A were added to the pellet, the extract incubated at 50 °C with gentle shaking and centrifuged. DNA in the supernatant was transferred and extraction was performed with an equal volume of phenol/chloroform/isoamyl alcohol (25:24:1). A second extraction with isopropanol was carried out and after centrifugation the DNA was recovered in the pellet. Three washes of ethanol were performed; the alcohol discarded and after drying DNA was redissolved in TE.

Plant materials sequenced in this study were acquired from the citrus germplasm banks of Palermo, Corsica and Instituto Valenciano de Investigaciones Agronomicas (IVIA) and from commercial nurseries, comprising a total of 34 citrus genotypes, including oranges, lemons, pummelos, grapefruits, limes, papedas, mandarins, kumquats, trifoliate, citrons, Australian limes, and a “Chinese box orange” (table 1). Genome sequences of Guanxi and Shatian pummelos and Mangshan and Huanglingmiao mandarins were obtained from the original publication of the draft genome of sweet orange (Xu et al. 2013).

### Genome Sequencing

Libraries were constructed using the Illumina TruSeq DNA Sample Prep standard protocol with some modifications. Briefly, 1 µg of high molecular weight genomic DNA was fragmented with a Covaris sonication device. Thereafter, DNA fragments were end-repaired and A-tailed. Adapters were then ligated through a 3′-thymine overhang. Finally, ligated fragments were amplified by polymerase chain reaction (PCR) (10 cycles). Library insert sizes ranged from 400 to 500 bp. The libraries were applied to an Illumina flowcell for cluster generation. Sequencing was performed on a HiSeq2000 instrument. Data generated consisted of 100-bp paired-end reads. Primary analysis of the data included quality control on the Illumina RTA sequence analysis pipeline.

### Nuclear-Chloroplast Homologous Regions

Homologous regions between chloroplast and nuclear genomes were excluded from the analysis. Chloroplast genome regions with nuclear homology were identified through Basic Local Alignment Search Tool (BLAST) (Altschul et al. 1990) analysis. BLAST hits with *e* value < 0.0001, size > 100 bp, and an identity > 90% were considered as homologous regions.

### Primary Data Processing

Raw sequences were first evaluated by a quality control tool, using the FastQC (http://www.bioinformatics.babraham.ac.uk/projects/fastqc/, last accessed April 4, 2015) tool to detect any potential undesirable artifact in data. Sample sequences were mapped against the *C. sinensis* chloroplast genome (Bausher et al. 2006). Chloroplast-mapped reads (average, 4.3 million/genome) and mean coverage (about 2,000) were relatively high, although the ratio of mapped reads against total reads was proportionally low because raw sequences also contained nuclear, plastid, and mitochondrial DNA.

The mapping procedure applied in this study is summarized as follows: 1) Paired-end reads were mapped using the Burrows–Wheeler Aligner software (Li and Durbin 2010), 2) reads with a low-quality alignments were then filtered out from mapping using Samtools (Li et al. 2009) using the default parameters, 3) duplicated reads were filtered out using the Picard tool (http://broadinstitute.github.io/picard/, last accessed April 4, 2015), 4) reads were realigned and mapped again around indels using GATK tool (McKenna et al. 2010), and 5) a final quality control was applied to all mapping files using the Qualimap tool (Garcia-Alcalde et al. 2012). Mapped reads with mapping quality ≥29 were filtered out. Only one hit was allowed for reads.

### Variant Calling

The final set of mapping files (VCF) was used to perform a multisample variant calling by using GATK (McKenna et al. 2010). The calling parameters were adjusted to obtain both single nucleotide polymorphism (SNP) and indel markers and haploid calls.

The resulting matrix, which contained the called genotype for every sample at every variant site, was filtered using the GATK recommended criteria (https://www.broadinstitute.org/gatk/guide/best-practices, last accessed April 4, 2015). Then, sample genotypes unsupported by at least 90% of covering reads were imputed as missing values. Variants within nuclear-chloroplast homologous regions were removed from matrix. Finally, SNVs and indels reported in this work were manually curated using the Integrative Genomics Viewer (IGV) software (Robinson et al. 2011) to visualize and confirm genomic reads.

### Phylogenetic Analysis

Only positions with SNVs that vary across the genomes studied were used in the analysis. Invariant positions were removed and positions in which at least one of the samples was different were used to build a concatenated in a single sequence per sample.

The phylogenetic tree was reconstructed using PhyML software (Guindon et al. 2005) using the SeaView graphical interface (Gouy et al. 2010) as well. The method used was a maximum-likelihood iterative model and a bootstrap of 1,000 repetitions was used to assess the reliability of the phylogeny reconstructed. In parallel, the phylogeny was also inferred from the set of selected markers using a Bayesian Markov chain Monte Carlo (MCMC) search (Yang and Rannala 1997) as implemented in the program MrBayes 3.1.4 (Huelsenbeck and Ronquist 2001), with two parallel runs of 5 million generations and ten chains each, using the general time reversible (GTR) model. This model was found to be optimal by the program JModeltest 2 (Posada and Crandall 1998; Darriba et al. 2012) using both the hierarchical LRT (Felsenstein 1988) and the Akaike information criterion (Akaike 1974). Convergence of the parallel runs was determined by examining the average standard deviation of split frequencies, which fell below 0.01. Clade support was assessed using the posterior probabilities from the Bayesian analysis and also using parsimony bootstrap analysis (Felsenstein 1985) with five random addition sequence replicates for each of 200 bootstrap replicates, holding a maximum of 100 trees per random addition sequence replicate.

### Clocklike Test

A relaxed molecular clock dating strategy was used to test whether the data presented a clocklike behavior or not. To test the clocklikeness of the data, The GTR model and the optimal parameter values from JModeltest 2 were specified in a Nexus format sequence file that was further analyzed using maximum likelihood in PAUP* 4.10 b (Wilgenbusch and Swofford 2003) in a heuristic search using five random addition sequence replicates. Then, the best tree obtained was used for carrying out an LRT in PAUP* of clocklike behavior of the data. Clock versus nonclock constraints were applied to the best tree, and the parameter values re-estimated with branch lengths to find the likelihood score of each constraint on the best tree.

### Estimation of Divergence Times

A strategy similar to Pfeil and Crisp (2008) was used for the calibration of the inferred phylogeny to estimate divergence times for the different citrus species. Molecular clock dating was carried out by applying the approach of minimum constraints using PL (Sanderson 2003), a widely used method that estimates uncertainty of the dates by taking into account uncertainty in the topology and branch lengths, allows calibrations with known speciation dates, accommodates rate differences among branches, and also includes the assumption of autocorrelation.

To calibrate the phylogenetic tree, estimations of dates of speciation events from literature were used (Pfeil and Crisp 2008). The *Severinia–Citrus* divergence (age of the root) has occurred approximately 13 Ma (range of uncertainty from 20 to 8 Ma), the *Poncirus*–tangerine divergence was about 6.6 Ma (from 11.6 to 3.2 Ma), and the separation between the two main citron branches has occurred approximately 7.1 Ma (from 11.8 to 3.7 Ma).

PL was combined with a Bayesian approach to estimate phylogenetic uncertainty (Welch and Bromham 2005) as follows: 100 trees were taken from the last 200 K generations of one of the MrBayes runs (arbitrarily chosen) and imported into r8s 1.8 (Sanderson 2003). Then, dates along with the corresponding CIs were estimated in PL using two strategies. In the first strategy, only the age of the root is fixed in a range that spans from 8 to 20 Ma (with increments of 1 Ma). A second strategy added two internal constraints: Australian citrons versus rest of citrus occurred between 3.7 and 11.8 Ma and the divergence between *Poncirus* and Pummelos/Mandarins occurred between 3.2 and 11.6 Ma. Cross-validation with the truncated Newton algorithm (Sanderson 2003) was used to find the optimal smoothing parameter, which resulted very close to 1. This second strategy was repeated using two other dating methods in r8s. First, Langley Fitch (LF) molecular clock (Langley and Fitch 1974) with the truncated Newton algorithm (Sanderson 2003) and second LF local clock with the Powell algorithm (Press et al. 1996) were used to test for differences that might be due to the assumptions implicit in the PL procedure.

### Selective Pressure Analysis

Signatures of natural selection were studied for every chloroplast gene located outside of the inverted repeats region. Selective pressures were computed with *codeml* tool from PAML package (Yang 2007). Three models were used to test every gene sequence: A fixed neutral ω (the Ka/Ks ratio, where Ka is the ratio of the number of nonsynonymous substitutions per nonsynonymous site and Ks the number of synonymous substitutions per synonymous site) model (ω = 1); a fixed estimated ω model, where “codeml” estimates the most likely ω for the whole tree; and a two-ratios, model for every node of the phylogenetic tree, where codeml estimates one ω for every possible clade and other ω for the rest of the tree. Log likelihood values of every model were compared against neutral model by means of an LRT, in order to test statistical significance. Finally, genes were considered to be under positive/negative selection at a certain clade when 1) its ω from two-ratio model was higher/lower than 1 (neutral selection) and 2) the log likelihood of its model was statistically different from the log likelihood of a fixed neutral model. To avoid potential convergence biases, those genes with few mutations were filtered out from selective pressure analysis.

### Analysis of Heteroplasmy

A position was defined as a heteroplasmic if the minor allele was present in at least 5 out of 1,000 reads. In case of doubt the surrounding genomic structure showed unambiguous interpretation through IGV (Robinson et al. 2011) visualization. Heteroplasmy analysis was done with custom scripts in R language (http://www.R-project.org, last accessed April 4, 2015). Visualization of tree in R and ancestral inference was done with the *ape* package (Popescu et al. 2012). A maternity test was implemented to check if minor allele in heteroplasmic loci could be produced due to a biparental heteroplasmy event (Moreira et al. 2002). The maternity test checks if minor heteroplasmic allele matches the alleles of a putative paternal parent at the same location. To avoid any potential bias, all known hybrids were not use as a putative paternal parent for other hybrids.

### Structural variation

SV analysis in the chloroplast genome was carried out by using two methods for SV prediction: DELLY (Rausch et al. 2012) and Breakdancer (Chen et al. 2009) and two methods specific for copy number variation (CNV) prediction (NMOPS [Klambauer et al. 2012] and Control-FREEC [Boeva et al. 2012]). In order to detect only highly reliable SVs, a consensus rule was used and only those detected by the four methods were selected for further analyses. This strategy reduced the false positive rate and guaranteed the discovery of high quality SVs. The final set of SVs was also manually curated through IGV visualization.

### DNA Extraction and PCR Analyses

PCR analyses were determined through real-time quantitative PCR, on a LightCycler 2.0 instrument (Roche) using the LightCycler FastStart DNA MasterPLUS SYBR Green I kit (Roche) essentially as described in Terol et al. (2015). Each individual PCR reaction contained 2 ng of genomic DNA extract from leaves using the DNeasy Plant Mini Kit (Qiagen) according to the manufacturer’s instructions. Cycling protocol consisted of 10 min at 95 °C for preincubation followed for 45 cycles of 10 s for denaturation, 10 s at 60 °C for annealing, and 20 s at 72 °C for extension. Specificity of the PCR reaction was assessed by the presence of a single peak in the dissociation curve after amplification and through size estimation of the amplified product. Specificity of PCR reaction was confirmed by direct Sanger sequencing of the PCR product. Primers used are listed in table 5.

**Table 5.** Specific Primers for the Determination of Heteroplasmic Positions.

Name	Frame	SNP Position	Sequence
C21826-F1	+	21826	5′-GCATCTTGGACTAGCCATCG-3′
C21826-R1	−		5′-ACCGTGGGCCATATTTCTCT-3′
C20848-F2	+	20848	5′-CCCGAATCTCAGCAATCACT-3′
C20848-R2	−		5′-TAACCCTTCGAACACAAGCA-3′
C69792-F1	+	69792	5′-GTTGCCCACTCAATCTGTTG-3′
C69792-R2	−		5′-AATCTGCCTTGCCTAGGAATC-3′

## Supplementary Material

Supplementary figures S1–S3 are available at *Molecular Biology and Evolution* online (http://www.mbe.oxfordjournals.org/).

Supplementary Data
