# Supplementary material for: A Phylogenetic Analysis of 34 Chloroplast Genomes Elucidates the Relationships between Wild and Domestic Species within the Genus Citrus
Source: Mol Biol Evol. 2015 Apr 14;32(8):2015–35. doi: 10.1093/molbev/msv082 (PMC4833069; doi:10.1093/molbev/msv082)
Supplement: Supplementary Data [file supp_32_8_2015__index.html]

A phylogenetic analysis of 34 chloroplast genomes elucidates the relationships between wild and domestic species within the genus Citrus — A Phylogenetic Analysis of 34 Chloroplast Genomes Elucidates the Relationships between Wild and Domestic Species within the Genus Citrus — A Phylogenetic Analysis of 34 Chloroplast Genomes Elucidates the Relationships between Wild and Domestic Species within the Genus Citrus — Supplementary Data 

# A Phylogenetic Analysis of 34 Chloroplast Genomes Elucidates the Relationships between Wild and Domestic Species within the Genus *Citrus*

## Supplementary Data

files

**Files in this Data Supplement:**

- Supplementary Data - tiff file
- Supplementary Data - tiff file
- Supplementary Data - docx file
- Supplementary Data - docx file
