# Supplementary figures and images for: A Phylogenetic Analysis of 34 Chloroplast Genomes Elucidates the Relationships between Wild and Domestic Species within the Genus Citrus
Source: Mol Biol Evol. 2015 Apr 14;32(8):2015–35. doi: 10.1093/molbev/msv082 (PMC4833069; doi:10.1093/molbev/msv082)

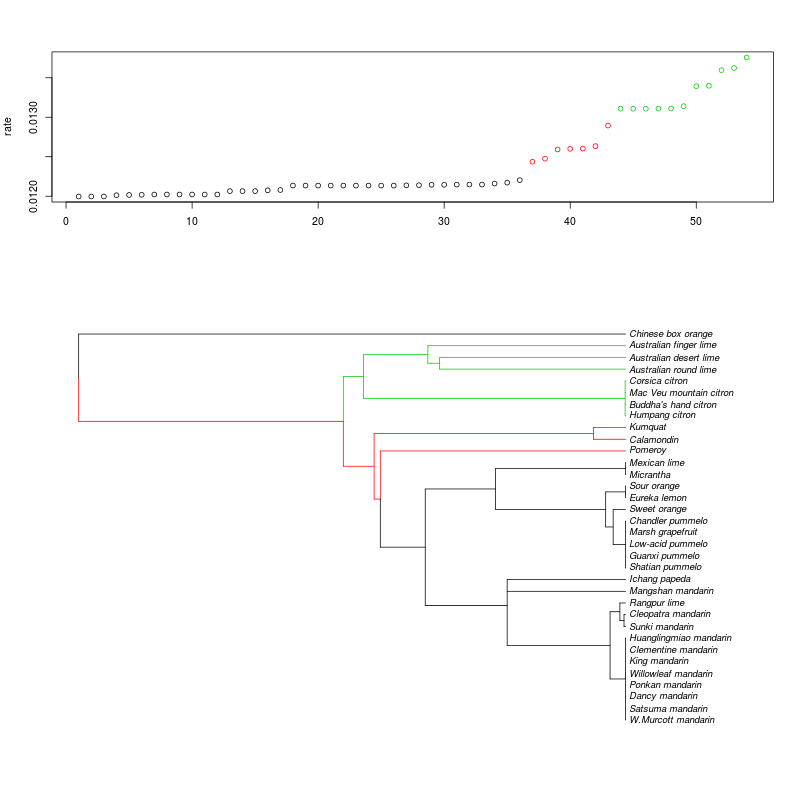

Supplement: Supplementary Data [file supp_msv082_Carbonell-Supplementary_Figure_1.tif]

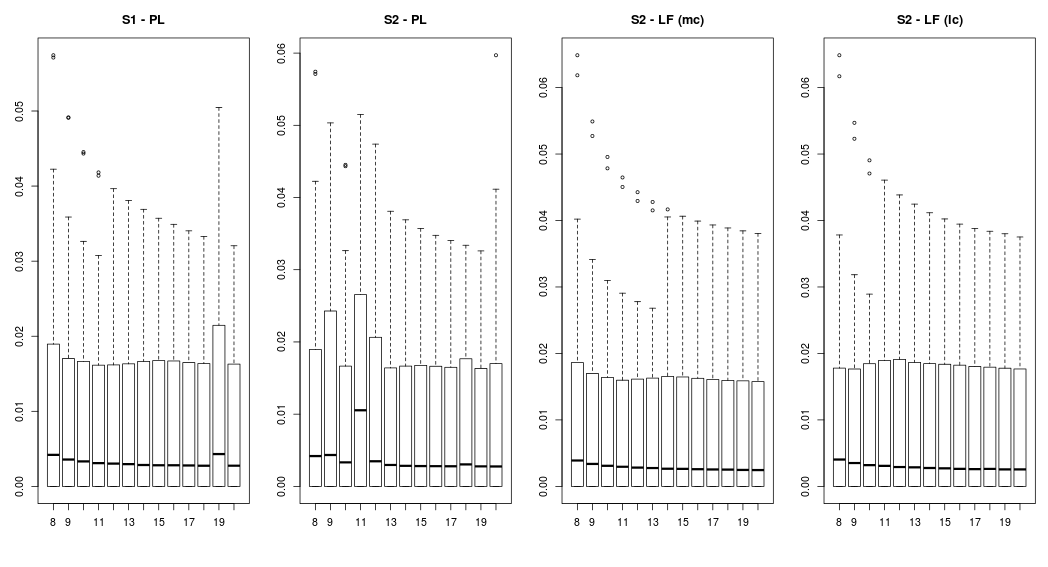

Supplement: Supplementary Data [file supp_msv082_Carbonell-Supplementary_Figure_2.tif]
