# Supplementary material for: A Phylogenetic Analysis of 34 Chloroplast Genomes Elucidates the Relationships between Wild and Domestic Species within the Genus Citrus
Source: Mol Biol Evol. 2015 Apr 14;32(8):2015–35. doi: 10.1093/molbev/msv082 (PMC4833069; doi:10.1093/molbev/msv082)
Supplement: Supplementary Data [file supp_msv082_Carbonell-Supplementary_Figure_3.docx]

|  | Position 21826 | Cultivar |
| --- | --- | --- |
| Maternal parent | 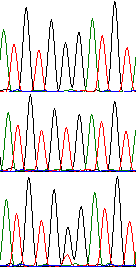 | *Mandarin* |
| Paternal parent |  | *Citron* |
| Hybrid |  | *C.limonia* |
|  | Position 20848 | Cultivar |
| Maternal parent | 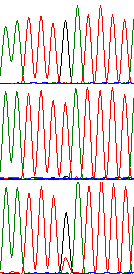 | *Mandarin* |
| Paternal parent |  | *Citron* |
| Hybrid |  | *C.limonia* |
|  | Position 69792 | Cultivar |
| Maternal parent | 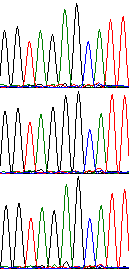 | *Micrantha* |
| Paternal parent |  | *Citron* |
| Hybrid |  | *C.aurantifolia* |
