# Supplementary material for: A Phylogenetic Analysis of 34 Chloroplast Genomes Elucidates the Relationships between Wild and Domestic Species within the Genus Citrus
Source: Mol Biol Evol. 2015 Apr 14;32(8):2015–35. doi: 10.1093/molbev/msv082 (PMC4833069; doi:10.1093/molbev/msv082)
Supplement: Supplementary Data [file supp_msv082_New_Microsoft_Office_Word_Document.docx]

**Supplementary figures.**

**Supplementary Figure 1.** Rates of branches obtained with the PL tree calibrated with 16 My for the root (upper part). Four classes have been defined and appear in different colours in the figure. Branches to which these rates, with the corresponding colour, have been assigned (lower part). Each branch was automatically assigned to a category by using a K-means classifier.

**Supplementary Figure 2.** Confidence intervals (CI) for nodes lengths obtained with the two strategies used (S1 only root date fixed and S2 constraints in branches allowed) with penalized likelihood, Langley and Fitch with molecular clock -LF (mc)- and also with local clock –LF(lc)-. The distribution of node CI length (normalized by corresponding root age) become stabilized from 13 to 18 Mya.

**Supplementary figure 3.** Validation of heteroplasmic positions in citrus hybrids. The figure shows sequencing chromatograms of PCR products amplified from three regions covering heteroplasmic positions detected by Illumina sequencing corresponding to: two heteroplasmic sites in the trio Mandarin (maternal parent) citron (paternal parent) and the hybrid *C. limonia* (upper and medium parts), and also in the trio Micrantha (maternal parent), citron (paternal parent) andthe hybrid *C. aurantifolia* (lower part).
